# The 2β Splice Variation Alters the Structure and Function of the Stromal Interaction Molecule Coiled-Coil Domains

**DOI:** 10.3390/ijms19113316

**Published:** 2018-10-25

**Authors:** Steve Chung, MengQi Zhang, Peter B. Stathopulos

**Affiliations:** Department of Physiology and Pharmacology, Schulich School of Medicine and Dentistry, The University of Western Ontario, 1151 Richmond Street, London, ON N6A 5C1, Canada; schun73@uwo.ca (S.C.); mzhan388@uwo.ca (M.Z.)

**Keywords:** stromal interaction molecule-1 (STIM1), stromal interaction molecule-2 (STIM2), store-operated calcium entry (SOCE), calcium signaling, alterative splicing, coiled-coil, structure, Fura-2

## Abstract

Stromal interaction molecule (STIM)-1 and -2 regulate agonist-induced and basal cytosolic calcium (Ca^2+^) levels after oligomerization and translocation to endoplasmic reticulum (ER)-plasma membrane (PM) junctions. At these junctions, the STIM cytosolic coiled-coil (CC) domains couple to PM Orai1 proteins and gate these Ca^2+^ release-activated Ca^2+^ (CRAC) channels, which facilitate store-operated Ca^2+^ entry (SOCE). Unlike STIM1 and STIM2, which are SOCE activators, the STIM2β splice variant contains an 8-residue insert located within the conserved CCs which inhibits SOCE. It remains unclear if the 2β insert further depotentiates weak STIM2 coupling to Orai1 or independently causes structural perturbations which prevent SOCE. Here, we use far-UV circular dichroism, light scattering, exposed hydrophobicity analysis, solution small angle X-ray scattering, and a chimeric STIM1/STIM2β functional assessment to provide insights into the molecular mechanism by which the 2β insert precludes SOCE activation. We find that the 2β insert reduces the overall α-helicity and enhances the exposed hydrophobicity of the STIM2 CC domains in the absence of a global conformational change. Remarkably, incorporation of the 2β insert into the STIM1 context not only affects the secondary structure and hydrophobicity as observed for STIM2, but also eliminates the more robust SOCE response mediated by STIM1. Collectively, our data show that the 2β insert directly precludes Orai1 channel activation by inducing structural perturbations in the STIM CC region.

## 1. Introduction

Store operated calcium (Ca^2+^) entry (SOCE) is a crucial signaling mechanism present in all eukaryotic cells. SOCE occurs after depletion of endoplasmic reticulum (ER) Ca^2+^ stores leads to activation of Ca^2+^ channels located on the plasma membrane (PM) [1]; further, this PM Ca^2+^ channel activation permits movement of extracellular Ca^2+^ down the steep concentration gradient into the cytosol, initiating downstream signaling involved in processes ranging from fertilization to cell death [2]. Stromal interaction molecules (STIM)s and Orai proteins are the key polypeptide macromolecules which mediate SOCE [3,4,5,6,7,8,9,10]. Most STIMs are localized in the ER membrane where they function as sensors of luminal Ca^2+^ levels. After decreases in stored ER luminal Ca^2+^, STIMs translocate to ER-PM junctions where they couple to PM Orai Ca^2+^ channel proteins [11,12,13,14]. The direct binding between STIM and Orai proteins opens the channel gate and permits movement of Ca^2+^ from the extracellular space into the cytosol [11,15,16,17,18,19].

**Figure 1:**
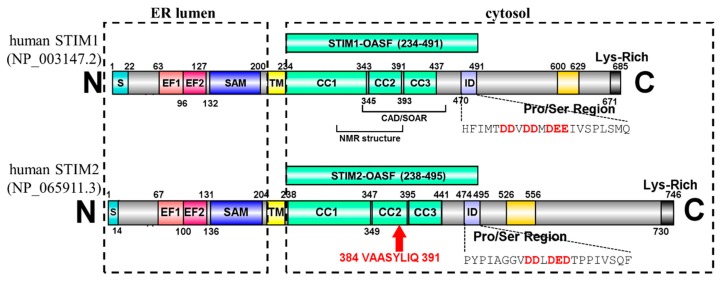
Human stromal interaction molecule (STIM1) and (STIM2) domain architecture. The residue ranges encompassing each conserved domain are indicated. The Orai-activating STIM fragment (OASF) constructs used in the present work are shown above the full-length architectures. The relative locations of the CRAC activating domain (CAD)/STIM-Orai activating region (SOAR) and the CC1-CC2 NMR structure fragment are indicated below the STIM1 architecture. The location of the 2β insert in the CC2 region of STIM2 is highlighted. The acidic residues found in the ID sequences are shown in red text. S, signal peptide; EF1, canonical EF-hand; EF2, non-canonical EF-hand; SAM, sterile α-motif; TM, transmembrane; CC1, coiled-coil-1; CC2, coiled-coil-2; CC3, coiled-coil-3; ID, inhibitory domain; N, amino terminus; C, carboxyl terminus.

Humans express two STIM homologues: STIM1 and STIM2 [20,21]. Both homologues are type I, single pass transmembrane (TM) proteins and contain a series of highly conserved domains on both sides of the TM (Figure 1). The N-terminal region consists of a canonical Ca^2+^ binding EF-hand, a non-conical EF-hand and a sterile α-motif (SAM) domain (i.e., collectively termed “EF-SAM”) which are oriented in the endoplasmic reticulum (ER) lumen [22,23]. After the single-pass TM domain, the cytosolic region contains three CCs (i.e., CC1-CC2-CC3), an inhibitory domain (ID), a Ser/Pro-rich region and a polybasic C-terminal region, each with a different role in the molecular mechanisms underlying SOCE. CC1 forms an extended α-helix spanning ~13 nm which bridges the bulk of the distance between the ER and PM [24]. CC2 together with CC3 establishes the minimal boundaries necessary for coupling to and gating Orai1 channels [16,18,19]. The acidic residue-rich ID region is thought to bind Ca^2+^ during Ca^2+^-dependent inactivation [25,26]. Together, the three CCs and ID fall within the Orai-activating STIM fragment (OASF) and are critical for mediating a compact to extended conformation integral to SOCE activation [27]. The Ser/Pro-rich region can be phosphorylated at several sites, promoting or inhibiting SOCE activation (recently reviewed in [28]). Since STIM1 is the primary regulator of SOCE [3,29,30], the specific conformational rearrangements which occur in the STIM1 conserved domains for SOCE activation are well-defined. Upon ER-luminal Ca^2+^ depletion, the EF-SAM region releases Ca^2+^, becomes destabilized, and undergoes a self-association (i.e., dimerization) [22,31]. This self-association leads to a rearrangement of the CC domains, resulting in a conformational extension [27,32], oligomerization [12,33,34], trapping of STIM1 at ER-PM junctions via interactions with PM phosphoinositides [35,36,37], and direct coupling to Orai1 proteins [16,17,18,19]. Ultimately, it is the binding between STIM1 and Orai1 which opens Orai1-composed Ca^2+^ channels [38].

In addition to the role in SOCE, STIM2 is an important mediator of basal Ca^2+^ homeostasis, as a large fraction of STIM2 actively gates Orai1 channels at resting ER Ca^2+^ levels, which is attributed to the weaker Ca^2+^ binding affinity of STIM2 compared to STIM1 [3,29,30]. STIM2 can also be alternatively spliced into the STIM2β isoform, also named STIM2.1 [39,40]. In contrast to STIM2, STIM2β contains an 8-residue insertion (i.e., VAASYLIQ) near the end of CC2 (Figure 1). The STIM2β isoform is incapable of eliciting SOCE, and it inhibits both STIM1- as well as STIM2-induced SOCE [39,40]. The mechanism of SOCE inhibition by STIM2β likely involves heterodimerization with STIM1, while the inability to induce SOCE may be related to disrupted interactions with Orai1 [39,40]. However, given that STIM2 couples weakly to Orai1 proteins in comparison to STIM1 [29,30,41,42], it remains unclear if the 2β insert further depotentiates STIM2 coupling to Orai1 or if this splice variation independently perturbs the STIM2 CC structure which precludes SOCE.

Here, we use far-UV circular dichroism (CD), light scattering, exposed hydrophobicity analysis and solution small angle X-ray scattering (SAXS) assessments of 2β incorporated into STIM1-OASF and STIM2-OASF proteins to elucidate the structural and biophysical consequences of the insertion. The entire OASF region was utilized in our work since each CC domain plays an important role in the activation mechanism of STIMs. Additionally, we engineered a full-length STIM1/STIM2β chimera to assess whether the 2β variation precludes STIM function in a homologue-dependent manner. We find that the 2β insert reduces the overall α-helicity, enhances the exposed hydrophobicity and increases the conformational sensitivity of OASF to Ca^2+^. Remarkably, incorporation of the 2β insert into the STIM1 context eliminates the robust SOCE mediated by STIM1. Collectively, our data show that the 2β insert directly precludes Orai1 channel activation by inducing structural perturbations in the STIM CC region.

## 2. Results

### 2.1. The 2β Insertion Decreases the α-Helicity of OASF

Using *Escherichia coli*, we expressed and purified recombinant human STIM1-OASF (i.e., residues 234–491; NCBI accession NP_003147.2), STIM1-2β-OASF (i.e., STIM1-OASF mutated to contain the VAASYLIQ (2β) insertion after G379), STIM2-OASF (i.e., residues 238–496; NCBI accession NP_065911.3) and STIM2-2β-OASF (i.e., residues 238–504 containing the naturally occurring VAASYLIQ insertion after E383; NCBI accession NP_001162589.1) to assess the effect of the 2β variation on the structural and biophysical properties of the STIM CC regions. All proteins were attainable at ~90–95% purity, as determined by Coomassie blue-stained SDS-PAGE (Figures S1 and S2).

**Figure 2:**
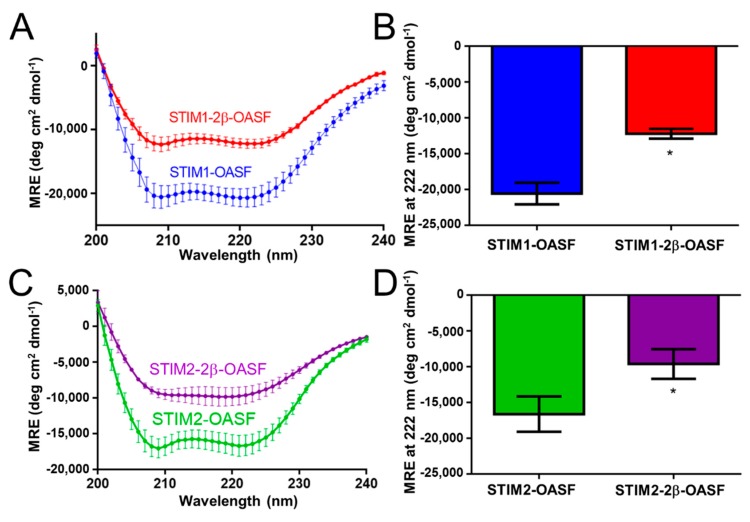
Secondary structure of STIM OASF proteins. (**A**) Far-UV CD spectra of STIM1-OASF and STIM1-2β-OASF. (**B**) Comparison of STIM1-OASF and STIM1-2β-OASF mean residue ellipticity (MRE) at 222 nm. (**C**) Far-UV CD spectra of STIM2-OASF and STIM2-2β-OASF. (**D**) Comparison of STIM2-OASF and STIM2-2β-OASF MRE at 222 nm. Spectra were acquired using 0.2 mg·mL^−1^ protein at 20 °C. Data are means ± SEM of *n* = 3 separate protein purifications. In (**B**,**D**), * *p* < 0.05 using Student’s *t*-test.

First, we used far-UV CD spectroscopy to assess the secondary structure of the STIM OASF domains with and without the 2β insert. The far-UV CD spectra showed that all STIM OASF homologues exhibit strong minima at 208 and 222 nm, consistent with the high levels of α-helicity characteristic of CC domains. However, incorporation of the 2β insert into the STIM1-OASF context resulted in a dramatic loss in the negative ellipticity at 208 and 222 nm (Figure 2A,B). Similarly, STIM2-OASF exhibited the α-helical minima expected at 208 and 222 nm, which became less intense in protein containing the 2β insert (Figure 2C,D). The less intense minima at these wavelengths indicate decreased α-helicity on a per residue basis for OASF proteins containing the 2β insert. Thus, the 2β region likely remains unstructured and may induce further unfolding perturbations in the OASF context.

### 2.2. The 2β Insert Reduces the Thermal Stability of STIM1-OASF

**Figure 3:**
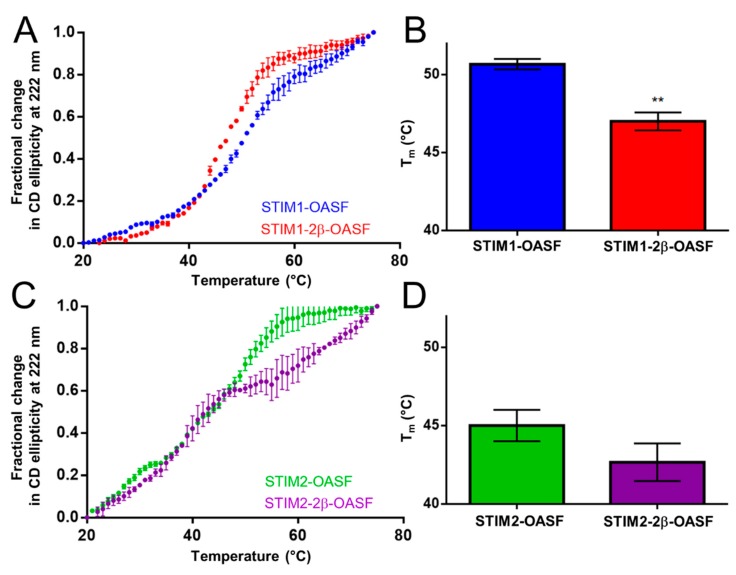
Thermal stability of STIM OASF proteins. (**A**) Thermal unfolding profile of STIM1-OASF and STIM1-2β-OASF. (**B**) Comparison of the STIM1-OASF and STIM1-2β-OASF T_m_ extracted from the thermal melts. (**C**) Thermal unfolding profile of STIM2-OASF and STIM2-2β-OASF. (**D**) Comparison of the STIM2-OASF and STIM2-2β-OASF T_m_ extracted from the thermal melts. Thermal melts were acquired using 0.2 mg·mL^−1^ protein. Data are means ± SEM of *n* = 3 separate protein purifications. In (**B**), ** *p* < 0.01 using Student’s *t*-test.

Having determined that the 2β insert reduces the overall level of α-helicity within OASF, we next assessed whether 2β affects stability by monitoring the change in far-UV CD signal at 222 nm as a function of temperature. The apparent midpoint of temperature denaturation (T_m_), defined as the temperature causing a 50% relative change in the CD signal, was taken as an indicator of thermal stability. In the STIM1-OASF context, the 2β insert induced a systematic leftward shift in the thermal melt profile (Figure 3A). The apparent T_m_ for STIM1-2β-OASF was 47.1 ± 0.1 °C compared to 50.5 ± 0.2 °C for STIM1-OASF (Figure 3B). This significant reduction in T_m_ shows that the 2β insert destabilizes the STIM1-OASF region.

The STIM2 OASF thermal melts exhibited less well-defined folded baselines compared to STIM1, reflective of poorer cooperativity in STIM2 CC domain unfolding. While the 2β insert did not induce a statistically significant change in T_m_, the first unfolding transition appeared complete at a much lower temperature (i.e., ~48 °C versus ~57 °C for STIM2-2β-OASF versus STIM2-OASF, respectively) (Figure 3C,D). Consequently, a second transition was more pronounced in STIM2-2β-OASF compared to any other OASF protein. This second transition was not related to an increased thermal stability, but instead represented the protein aggregation and precipitation phase which started at a lower temperature for STIM2-2β-OASF compared to STIM2-OASF. It is noteworthy that STIM2-OASF is markedly less stable than STIM1-OASF (i.e., ΔT_m_ = ~−6 °C) (Figure 3A,C) which may be associated with its weaker ability to activate SOCE [29,30,41,42]. Collectively, the thermal stability data show that the 2β insert destabilizes STIM1-OASF and alters the thermal unfolding profile for STIM2-OASF such that the unfolding transition is completed at a lower temperature.

### 2.3. The 2β Insert Exacerbates Ca^2+^-Induced Hydrophobic Exposure of OASF

**Figure 4:**
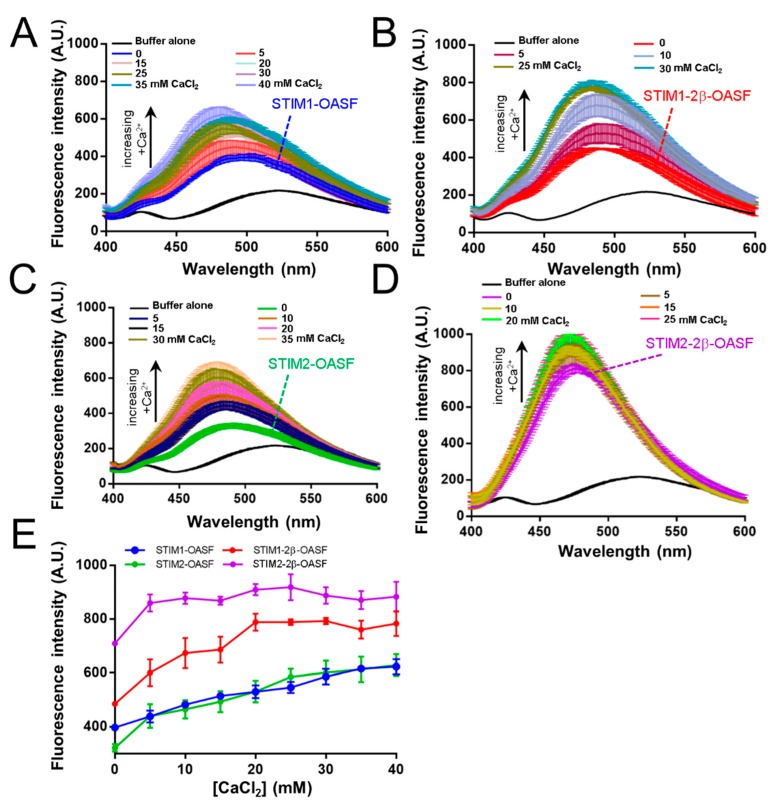
Extrinsic hydrophobicity of STIM OASF proteins. ANS fluorescence emission spectra of STIM1-OASF (**A**), STIM1-2β-OASF (**B**), STIM2-OASF (**C**), and STIM2-2β-OASF (**D**) as a function of increasing CaCl_2_ concentrations. In (**A**–**D**), the black spectra represent the buffer control scans. Spectra were acquired using 0.14 mg·mL^−1^ protein, 50 μM ANS and an excitation wavelength of 372 nm at 35 °C. (**E**) Change in the maximum ANS fluorescence intensity for STIM1-OASF (blue), STIM1-2β-OASF (red), STIM2-OASF (green) and STIM2-2β-OASF (purple) plotted as a function of CaCl_2_ concentration. Data are means ± SEM of *n* = 3 separate protein purifications.

Previous work showed that STIM1-OASF contains potential Ca^2+^ binding site(s) within an ID region of the cytosolic domains (Figure 1). The ID contains a high frequency of negatively charged residues, and interactions of the ID with cytosolic Ca^2+^ may perturb CC associations key for mediating SOCE. This cluster of negatively charged residues is mostly conserved in STIM2 (Figure 1). Having determined that the 2β insert decreases the folding and apparent stability of OASF, we next assessed whether 2β alters the effects of Ca^2+^ on OASF conformation. We used 8-anilinonaphthalene-1-sulfonic acid (ANS) binding to probe the relative solvent-exposed hydrophobicity of our OASF proteins with and without Ca^2+^. In the absence of Ca^2+^, STIM1-2β-OASF displayed a slightly higher ANS binding-induced fluorescence compared to STIM1-OASF (Figure 4A,B). The trend of higher ANS binding-induced fluorescence was also observed for STIM2-2β-OASF compared to STIM2-OASF (Figure 4C,D); however, the 2β variation much more markedly enhanced ANS binding in the STIM2 context compared to the STIM1 context (Figure 4A,C) which may be related to the lower stability for STIM2-OASF (see above).

For all OASF proteins, the ANS fluorescence intensity emission maxima increased and shifted to lower wavelengths, indicative of greater exposed hydrophobicity in the presence of Ca^2+^ (Figure 4A–D). In the high Ca^2+^ concentration regime tested, STIM1-2β-OASF and STIM2-2β-OASF proteins showed saturable ANS binding at lower Ca^2+^ concentrations compared to the proteins without 2β (Figure 4E). We believe this apparent saturable ANS binding is not due to higher Ca^2+^ binding affinity but, rather, the observation reflects the greater exposed hydrophobicity of OASF upon 2β incorporation which may be priming OASF into the inhibitory state promoted by Ca^2+^ binding.

Taken together, our ANS data suggest that the 2β insert increases the exposed hydrophobicity of STIM1-OASF and STIM2-OASF, and this conformational change alters the ANS binding properties of the fragment such that the maximal change in conformation is attained at lower Ca^2+^ concentrations. Additionally, the conformational sensitivity of all OASF proteins to CaCl_2_ supports the existence of Ca^2+^ binding sites for both STIM1 and STIM2. Importantly, the higher ANS fluorescence observed for proteins containing the 2β insert is consistent with the decreased folding and stability described above.

### 2.4. Ca^2+^ Minimally Affects the α-Helicity of the OASF Domains with or without 2β

Given our ANS data indicating that Ca^2+^ induces a conformational change in all OASF proteins, we next assessed whether the perturbations are coupled to changes in secondary structure. Again, we used far-UV CD spectroscopy to assess α-helicity. The addition of 25 mM CaCl_2_ to the STIM1-OASF and STIM1-2β-OASF proteins resulted in CD spectra which showed a trend toward less negative ellipticity at both the 208 and 222 nm minima; however, this apparent loss in α-helicity was diminutive and not statistically significant (Figure S3A–D). Similarly, STIM2-OASF and STIM2-2β-OASF showed no statistically significant change in negative ellipticity in the presence of 25 mM CaCl_2_ (Figure S3E–H). Collectively, the data show that the Ca^2+^-induced conformational changes observed via ANS binding in all OASF proteins are not linked with prominent changes in α-helicity.

### 2.5. Ca^2+^ Thermally Destabilizes STIM1-OASF and STIM1-2β-OASF

Next, we monitored changes in far-UV CD signal at 222 nm as a function of temperature to assess whether Ca^2+^ affects the thermal stability of the OASF proteins. Indeed, both STIM1-OASF and STIM1-2β-OASF exhibited significantly decreased thermal stabilities in the presence of 25 mM CaCl_2_ (i.e., T_m_ = 47.1 ± 0.2 °C and 50.5 ± 0.2 °C for STIM1-OASF in the presence and absence of CaCl_2_, respectively; T_m_ = 42.3 ± 0.6 °C and 46.8 ± 0.2 °C for STIM1-2β-OASF in the presence and absence of CaCl_2_, respectively) (Figure S4A–D). In contrast, the presence of 25 mM CaCl_2_ did not significantly alter the STIM2-OASF and STIM2-2β-OASF T_m_ values (i.e., T_m_ = 45.0 ± 0.4 °C and 46.6 ± 0.5 °C for STIM2-OASF in the presence and absence of CaCl_2_, respectively; T_m_ = 41.2 ± 0.9 °C and 42.6 ± 0.9 °C for STIM2-2β-OASF in the presence and absence of CaCl_2_, respectively) (Figure S4E–H). Therefore, our data suggest that Ca^2+^ significantly destabilizes STIM1-OASF and STIM1-2β-OASF, but not STIM2-OASF or STIM2-2β-OASF. We believe these homologue-specific differences reflect the lower α-helicity and thermal stability observed for STIM2-OASF and STIM2-2β-OASF compared to the STIM1 counterparts even in the absence of Ca^2+^, resulting in a more tempered Ca^2+^-induced destabilization for the already relatively unstable STIM2-OASF and STIM2-2β-OASF proteins.

### 2.6. Ca^2+^ Increases the Aggregation Propensity of OASF

**Figure 5:**
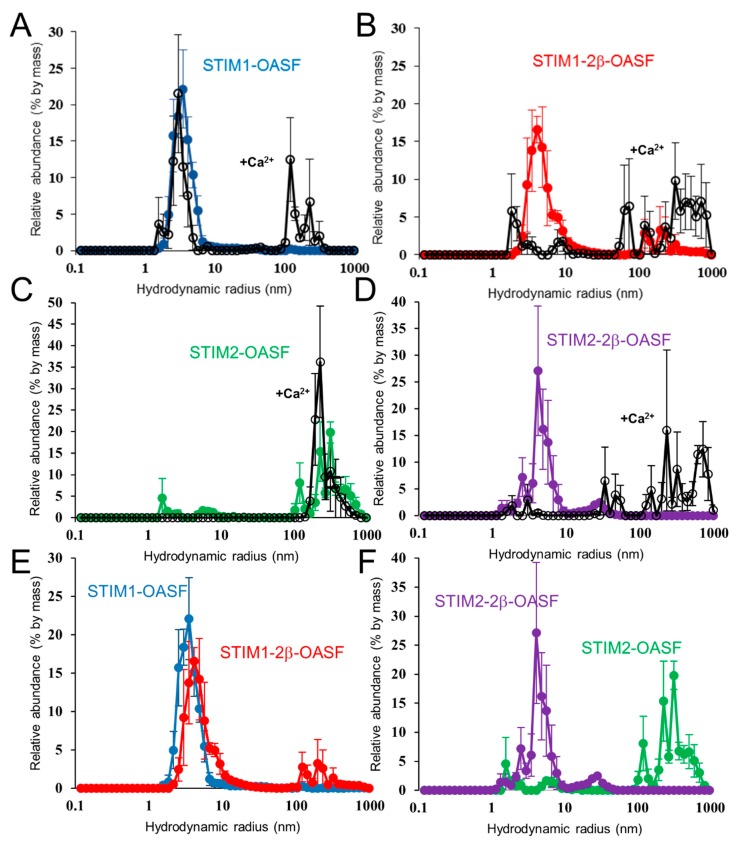
Hydrodynamic size distributions of STIM OASF proteins. (**A**) Distribution of hydrodynamic radii for STIM1-OASF. (**B**) Distribution of hydrodynamic radii for STIM1-2β-OASF. (**C**) Distribution of hydrodynamic radii for STIM2-OASF. (**D**) Distribution of hydrodynamic radii for STIM2-2β-OASF. In (**A**–**D**), the sizes are derived from the regularization deconvolution of the autocorrelation functions acquired using 0.5 mg·mL^−1^ protein at 35 °C in the absence (colored circles) and presence of 25 mM CaCl_2_ (open circles). (**E**) Direct comparison of the STIM1-OASF and STIM1-2β-OASF size distributions in the absence of CaCl_2_. (**F**) Direct comparison of the STIM2-OASF and STIM2-2β-OASF size distributions in the absence of CaCl_2_.

As another assessment of conformation, we evaluated the oligomerization propensity of the OASF proteins by dynamic light scattering (DLS). These DLS experiments were performed following centrifugation to pellet and remove any pre-aggregated protein (see Section 4). In the absence of Ca^2+^, STIM1-OASF and STIM1-2β-OASF samples showed major distributions (i.e., >90% by mass) of hydrodynamic radii (R_h_) centered at ~3.5 and 4.1 nm, respectively. Upon addition of 25 mM CaCl_2_, both STIM1-OASF and STIM1-2β-OASF samples exhibited a large fraction (i.e., >25% by mass) of higher order oligomers with R_h_ > 100 nm (Figure 5A,B). Interestingly, STIM2-OASF was found in a largely aggregated state with most particles exhibiting R_h_ of >100 nm (i.e., >90% by mass) (Figure 5C). In contrast to STIM2-OASF, STIM2-2β-OASF showed a lower propensity for oligomerization with most particles exhibiting an R_h_ of ~4.1 nm, resembling the distribution observed for STIM1-2β-OASF (Figure 5D). Upon addition of 25 mM CaCl_2_, STIM2-OASF maintained its predominantly aggregated state, while the majority of the STIM2-2β-OASF protein was converted to larger aggregates with R_h_ >100 nm (Figure 5C,D).

Taken together, the DLS data show that Ca^2+^ induces and/or favors higher order oligomerization of all OASF proteins, in-line with the markedly increased ANS binding caused by Ca^2+^ (see above). Furthermore, STIM1-2β-OASF and STIM2-2β-OASF adopt conformations with similar oligomerization propensities in the presence and absence of Ca^2+^. This effect on conformation is most palpable in the STIM2 context where STIM2-OASF is largely oligomerized whereas STIM2-2β-OASF predominantly shows much smaller R_h_ values which closely match STIM1-2β-OASF (Figure 5E,F).

### 2.7. The 2β Insertion Does Not Alter OASF Dimer Quaternary Structure

Having discovered that the 2β insert promotes a similar distribution of particle sizes in the STIM1-2β-OASF and STIM2-2β-OASF contexts, we next probed the quaternary structure of the assembling building blocks using size exclusion chromatography with in-line multi-angle light scattering (SEC-MALS). In contrast to DLS, which was performed at 35 °C, SEC-MALS was performed at ~10 °C to stabilize the lower order quaternary structures (see Methods). The SEC-MALS-determined molecular weights of all the OASF proteins were indicative of a dimeric state in both the presence and absence of 25 mM CaCl_2_ (Figure S5A–D). Consistent with these constant molecular weights, the elution volumes of all proteins were unaltered in the presence of CaCl_2_. Nevertheless, the STIM1-2β-OASF protein showed an earlier elution time compared to STIM1-OASF, and this decreased elution volume closely resembled those of STIM2-OASF and STIM2-2β-OASF (Table 1).

We previously showed that STIM1-OASF shifts to earlier elution times without an accompanying change in molecular weight when transitioning to a more extended conformation [27]. Therefore, the SEC-MALS data revealed that all OASF proteins maintain a dimeric stoichiometry in the presence and absence of Ca^2+^; however, STIM1-OASF elutes at larger elution volumes suggesting a more compact OASF conformation compared to STIM1-2β-OASF, STIM2-OASF and STIM2-2β-OASF.

### 2.8. The 2β Insertion Causes Local Changes in OASF Structure

Given that the 2β insertion decreases the level of α-helicity without altering the assembling stoichiometry of STIM1-OASF or STIM2-OASF, we next assessed whether the 2β variation induces global structural changes to the domain using SAXS. SAXS was acquired at relatively low concentrations (i.e., ~1 mg·mL^−1^) to limit protein aggregation; however, the low concentration yielded noisy scattering profiles, which were particularly evident at higher S values. Nevertheless, STIM1-OASF provided the SAXS profile with the least variability at higher S values (Figure S6A). From this data, the radius of gyration (R_g_) was determined to be 2.7 nm using a Guinier plot (Figure S6B). The maximum particle diameter (D_max_) estimated from the Porod distance distribution plot [P(*r*)] was 11.4 nm (Figure S6C). Similar analyses for STIM1-2β-OASF revealed an R_g_ and D_max_ of 2.9 and 11.1 nm, respectively (Figure S6D–F). In comparison, the STIM2-OASF and STIM2-2β-OASF proteins showed R_g_ values of 3.0 and 3.4 nm, respectively, and D_max_ values of 11.4 and 13.2 nm, respectively (Figure S7A–F).

**Figure 6:**
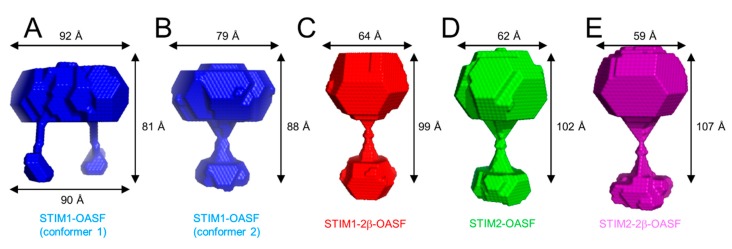
SAXS-derived bead models of STIM OASF proteins. DAMMIF-derived bead models of STIM1-OASF (conformation 1) (**A**), STIM1-OASF (conformation 2) (**B**), STIM1-2β-OASF (**C**), STIM2-OASF (**D**), and STIM2-2β-OASF (**E**). The reconstructed scattering profiles based on the STIM1-OASF, STIM1-2β-OASF, STIM2-OASF and STIM2-2β-OASF DAMMIF models are plotted in Figures S6A,D and S7A,D, respectively.

Next, we used the scattering profiles and D_max_ to generate low resolution bead models using DAMMIF [43]. Interestingly, for the STIM1-OASF data, two possible conformations were reconstructed. One conformation showed a single extension, presumably corresponding to interacting CC1 domains, while the second conformation showed two separated CC1 domains (Figure 6A,B). Reassuringly, the model showing separated CC1 regions bears a striking resemblance to the SAXS model reconstructed from STIM1 protein fragment encompassing residues 254–504 [24]. The bead models for the STIM1-2β-OASF, STIM2-OASF and STIM2-2β-OASF all exhibited very similar conformations as the STIM1-OASF model with the single extension (Figure 6C–E).

Overall, the SAXS data suggest that the 2β insert shifts OASF structure to slightly larger R_g_ in both the STIM1-OASF and STIM2-OASF contexts; however, no discernable trend was observed in D_max_, suggesting that the structural changes caused by the 2β variation may be more localized. Consistent with this notion, the bead models for all OASF proteins showed similar accessible global conformations with or without the 2β insertion.

### 2.9. The 2β Insertion Inhibits OASF Function Independent of the STIM2 Context

The 2β insertion has only been identified in STIM2 homologues, and the inability of STIM2β to induce SOCE has been well established. Even without the 2β variation, STIM2 couples to Orai1 and activates SOCE less efficiently in comparison to STIM1 [29,30,41,42]. Given our data showing decreased α-helicity, enhanced surface-exposed hydrophobicity and similar global conformations for both STIM1-2β-OASF and STIM2-2β-OASF compared to STIM1-OASF and STIM2-OASF, respectively, the 2β insert appears to congruently perturb OASF biophysical properties of both STIM1 and STIM2. Nevertheless, to determine whether the 2β insert directly prevents SOCE rather than further depotentiating the already weak STIM2-mediated SOCE activation, we created a full-length STIM1/STIM2β chimera. This chimera was comprised of full-length STIM1 with monomeric cherry fluorescent protein (mCh) and the 2β variation incorporated immediately downstream of the ER signal peptide and G379 within OASF, respectively (i.e., mCh-STIM1-2β).

**Figure 7:**
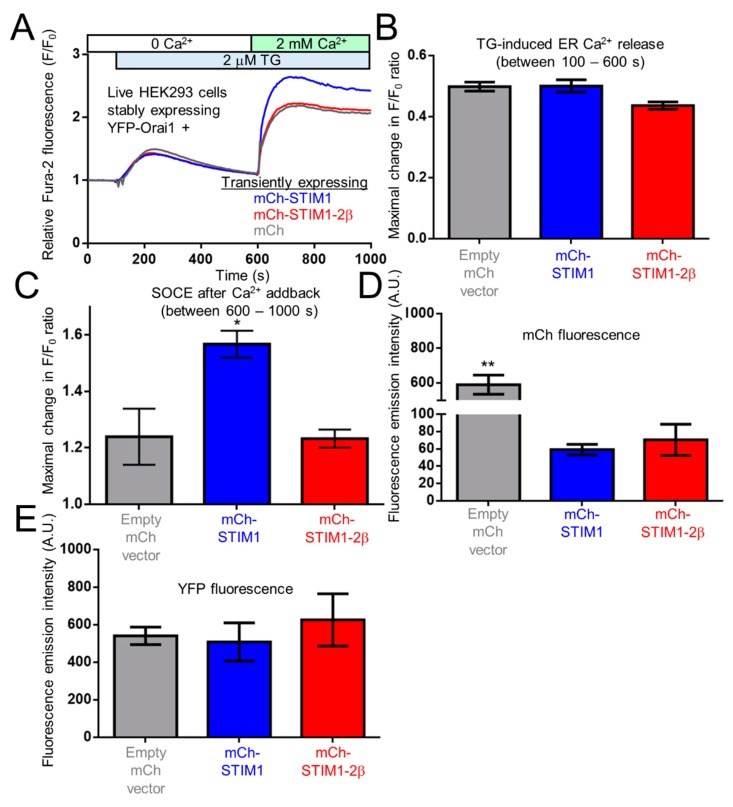
Fura-2 cytosolic Ca^2+^ assessment of full-length STIM1 and STIM1-2β function in SOCE. (**A**) Representative Fura-2 ratiometric fluorescence traces reporting relative changes in cytosolic Ca^2+^ of mCh-STIM1 (blue), mCh-STIM1-2β insert (red) and empty mCh-vector (grey) expressing HEK293 cells also stably expressing yellow fluorescence protein (YFP)-Orai1. Cells were initially bathed in Ca^2+^-free buffer. The relative change in Fura-2 fluorescence was monitored after 2 μM TG and subsequently 2 mM CaCl_2_ additions to the external medium. (**B**) Maximal ER Ca^2+^ release (i.e., F/F_0_ taken after TG addition in 0 mM external CaCl_2_. (**C**) Maximal SOCE taken after addition of 2 mM CaCl_2_ to the external medium. (**D**) Maximum mCh fluorescence emission of cell suspensions using an excitation wavelength of 565 nm. (**E**) Maximum YFP fluorescence emission of cell suspensions using an excitation wavelength of 490 nm. Data in (**B**–**E**) are means ± SEM of *n* = 4 separate transfections. In (**C**) * *p* < 0.05 and (**D**) ** *p* < 0.01 using One-way ANOVA and Tukey’s multiple comparisons test.

Subsequently, HEK293 cells stably expressing yellow fluorescence protein (YFP) tagged Orai1 (YFP-Orai1) were transfected with mCh-vector, mCh-STIM1 or mCh-STIM1-2β. After loading these transfected cells with Fura-2, SOCE was assessed using ratiometric fluorescence spectroscopy. Cells were initially bathed in HBSS medium free of Ca^2+^. Upon addition of thapsigargin (TG), the transient release of Ca^2+^ from the ER was not significantly different between the empty mCh-vector, mCh-STIM1 and mCh-STIM-2β expressing groups (Figure 7A,B). After 500 s of passive ER Ca^2+^ store depletion, 2 mM of Ca^2+^ was supplemented into the cell bathing medium. As expected, cells expressing mCh-STIM1 showed a significantly higher level of SOCE compared to the empty mCh-vector expressing cells. In contrast, when Ca^2+^ was added to cells expressing mCh-STIM1-2β, the maximal increase in the Fura-2 ratio was not significantly different than the empty mCh-vector expressing cells (Figure 7A,C).

To ensure that the differences in SOCE mediated by mCh-STIM1 and mCh-STIM1-2β were not due to differences in expression levels, we obtained mCh and YFP fluorescence emission spectra of the cell suspensions used in the Fura-2 measurements. As expected, maximal mCh-STIM1 and mCh-STIM1-2β fluorescence intensities were not significantly different from one another. The mCh-vector expressing cells showed higher mCh fluorescence intensity maxima since mCh was not targeted to the ER (Figure 7D). Maximum YFP fluorescence levels were not different between the three groups tested (Figure 7E).

Collectively, these results show that similar to STIM2, incorporating the 2β insert into OASF of full length STIM1 precludes SOCE activation, suggesting that the VAASYLIQ (i.e., 2β) insertion causes a dysfunctional OASF region, independent of a homologue-specific context.

## 3. Discussion

The CAD domain, also identified as the STIM-Orai activating region (SOAR), is primarily composed of CC2 and CC3 and encompasses all the protein machinery required to activate Orai1-composed Ca^2+^ channels [16,18,19]. Nevertheless, CC1 is essential for the conformational rearrangement which extends the cytosolic domains to bridge the distance between the ER and PM and permits direct contact between CAD and Orai1 [24,27,32]. Thus, the entire OASF region is critical in SOCE regulation. Indeed, heritable mutations in the STIM1 CAD as well as CC1 regions lead to disease. The R304W mutation in CC1 of STIM1 is an inherited mutation which constitutively activates SOCE and leads to the autosomal dominant Stormorken syndrome [44,45]. Symptoms of this syndrome include thrombocytopenia, muscle fatigue, asplenia, miosis, migraine, dyslexia and ichthyosis. This mutation causes an α-helical extension in CC1, conformational extension of OASF, increased self-association of the domain, and concomitant exposure of the CAD region [46]. Collectively, these conformational changes result in constitutive activation of Orai1 channels. In contrast, the homozygous inheritance of the R429C mutation in CC3 of STIM1 results in severe immunodeficiency, characterized by impairment of T-cell activation, autoimmune cytopenia, lymphoproliferation and prolonged diarrhea [47]. This mutation results in decreased α-helicity, destabilization of OASF, constitutive conformational extension of OASF, impairment of cytosolic domain oligomerization, and disruption of STIM1-Orai1 interactions [48]. Collectively, these conformational changes culminate in the inability of STIM1 to activate Orai1 channels. Cells have also evolved the ability to naturally modify residues in the OASF region as a normal mechanism of SOCE regulation. For instance, phosphorylation of T389 located in CC2 inhibits the ability of STIM1 to activate SOCE [49]. The inhibitory effect of this phosphorylation can be artificially mimicked through incorporation of a T389E mutation [50]. Incorporation of the phosphomimetic into CC2 thermally stabilizes OASF, and molecular dynamics simulations suggest that phosphorylation or the phosphomimetic extends the α-helix of CC2 [50].

Alterative splicing is another natural mechanism of SOCE regulation. For example, the retention of the 8-amino acid residue stretch (i.e., 2β insert) in the CAD domain of STIM2 gives rise to an isoform incapable of activating SOCE. Previous studies probing the mechanisms of STIM2β function have yielded some conflicting observations. Miederer and co-workers demonstrated that knockdown or overexpression of STIM2β does not alter basal Ca^2+^ levels in the cytosol or ER, but enhances or inhibits the rates and maximal plateau levels of SOCE, respectively [39]. Additionally, this group showed that STIM2β retains the ability to co-localize with Orai1 at ER-PM junctions, but close contact between STIM2β and Orai1 is abolished. Miederer and co-workers also assessed the effects of STIM2β and STIM1 co-expression, finding suppressed SOCE despite the STIM1 expression. Intriguingly, the ability of STIM2β to act as an inhibitor depended on the presence of Ca^2+^ in the cytosol and was loosely attributed to calmodulin (CaM) binding. Finally, Miederer et al. suggested that the 2β insertion could lead to perturbed dimer formation due to electrostatic repulsion.

In a second independent study, Rana and co-workers showed that STIM2β overexpression results in a small decrease in basal cytosolic and ER Ca^2+^ levels along with a strong inhibition of SOCE [40]. This group showed that STIM2β only weakly forms puncta, and that the ability to recruit Orai1 at these puncta, as well as the close contact between STIM2β CAD and Orai1, is severely impaired. Rana et al., also found that STIM2β and STIM1 CAD were able to form heterodimers which were necessary for SOCE inhibition. Intriguingly, it was demonstrated that STIM2β CAD domain tethering to the constitutively active Orai1-V102C channels inhibited SOCE. Thus, the authors suggest two possible mechanisms for SOCE inhibition: passive and active. In the passive model, STIM2β may sterically occlude other STIM molecules from binding to Orai1; in the active model, STIM2β may directly send an inhibitory signal to Orai1.

Finally, Zhou and co-workers found that homodimeric STIM2β CAD is unable to interact with Orai1, while heterodimers of STIM2β and STIM1 CAD indeed interact with Orai1 and fully activate channels [51]. Rather, this group suggested that the heterodimers prevent crosslinking of different Orai1 channels, thereby suppressing SOCE during oscillatory signaling. The mechanism underlying the defective homodimer interaction with Orai1 was proposed to be mediated by a helical extension of CC2.

Here, our structural studies using far-UV CD spectroscopy show that 2β decreases the α-helicity and destabilizes OASF. Additionally, our ANS binding experiments reveal that 2β increases the exposed hydrophobicity of OASF and the maximum structural response induced by high concentrations of Ca^2+^. Based on our SEC-MALS data, these changes in secondary structure and hydrophobic exposure do not affect the assembling dimer structure of OASF. Further, our SAXS analyses suggest that similar overall shapes are accessible by OASF proteins with and without the 2β insert. Finally, using Fura-2 cytosolic Ca^2+^ measurements and a full-length STIM1/STIM2β chimera, we discovered that the 2β insert abolishes STIM1 activation, as previously observed for STIM2β.

Our work on OASF argues against the notion that the 2β insert disrupts dimerization of STIM molecules, as the SEC-MALS data strictly show dimers for both STIM2-2β-OASF and STIM1-2β-OASF. Additionally, rather than extending the CC2 helix, our far-UV CD data show that the 2β insert decreases the overall α-helicity of OASF. Interestingly, we found that the 2β insert enhances the exposed hydrophobicity of OASF such that the Ca^2+^-induced conformational change is saturable. Previous work has identified the series of negatively charged residues in the ID as a putative Ca^2+^ binding site which promotes Ca^2+^-binding-dependent inactivation [25,26]; this inactivation was proposed to be due to a conformational change within the STIM1 cytosolic domain, impacting the interaction with Orai1. Our work suggests that Ca^2+^ can directly elicit a conformational change in OASF without requiring CaM, and that the 2β incorporation promotes maximal structural changes at lower Ca^2+^ levels. While past 2β studies focused on the role of CAD in SOCE inhibition, our SEC and SAXS data show that the 2β variation does not impair another important step in STIM activation: the conformational extension of the cytosolic domains mediated by CC1. Thus, SOCE inhibition is unlikely to be a result of a defective capability for extension, and trapping of extended STIM2β at ER-PM junctions should be possible, consistent with previously published work [39,40].

It is important to note that, although the DAMMIF bead models indicate similar accessible global shapes for all OASF proteins, visual inspection of the P(*r*) plots suggests that STIM1-2β-OASF may have an increased globularity compared to the other OASF proteins, as indicated by the disappearance of the shoulder at high *r* [52] (Figures S6C,F and S7C,F). Structurally, this increased apparent globularity may be caused by more dynamic and less stable CC interactions within STIM1 CAD induced by the 2β insertion. Nevertheless, the discrepancy between the DAMMIF model and qualitative assessment of the P(*r*) plot highlights the need for better quality SAXS data and/or high resolution structural data on the full cytosolic region of STIMs with and without the 2β insert.

**Figure 8:**
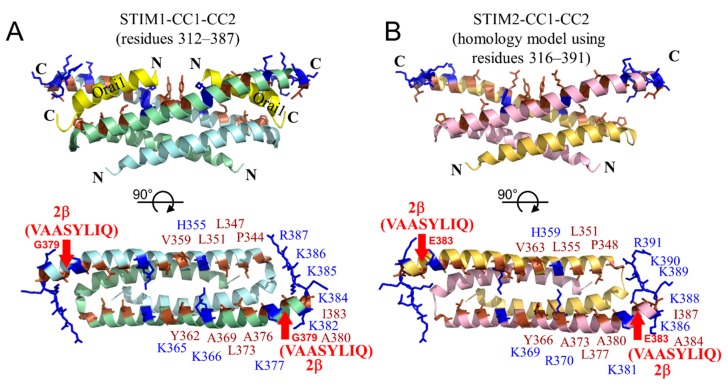
STIM CC1-CC2 structures identifying the location of the 2β variations. (**A**) Cartoon view of the backbone atoms making up the CC1 and CC2 helices in the dimeric human STIM1 CC1-CC2 solution structure. The location of the two human Orai1 C-terminal helices (yellow ribbons) are indicated (top view). The hydrophobic (brown sticks) and positively charged (blue sticks) residues within the CC2 helices making up the Orai1 C-terminal helix binding pocket are indicated and labeled (bottom view). (**B**) Cartoon view of the backbone atoms making the CC1 and CC2 helices in the dimeric human STIM2 homology model. The hydrophobic and positively charged residues making up the putative Orai1 C-terminal helix binding sites are indicated at top and bottom views. In (**A**,**B**), the relative locations of the 2β variations within CC2 are shown (red arrows). The STIM2 homology model was created from the STIM1 CC1-CC2 solution structural coordinates (2MAK.pdb) in Modeller [54]. Structure images were rendered using PyMOL.

How does 2β preclude activation of Orai1 channels? Our data suggest that reduced α-helicity caused by 2β would directly affect the coupling mechanism with the Orai1 C-terminal domains. We previously solved the solution nuclear magnetic resonance (NMR) dimer structure of human STIM1 CC1-CC2 (i.e., residues 312–387) bound to two human Orai1 C-terminal peptides (i.e., residues 273–293) [53]. The CC2 helices from each STIM1 monomer, which comprise approximately half of CAD, forms a hydrophobic cleft surrounded by an electropositive rim. Both the hydrophobic and electrostatic interactions were found to be important for interactions with the largely electronegative Orai1 C-terminal peptides [53]. Incorporation of the 2β insert (i.e., VAASYLIQ) into CC2 beginning at G379 would undoubtedly perturb the orientation of the positively charged residue-rich region located C-terminal to the insert and likely alter the precise positions of the hydrophobic residues N-terminal to the insert (Figure 8A). Mutations of either the positively charged or hydrophobic residues were previously found to decrease SOCE in experiments using full-length STIM1 [53]. The nature of the inserted sequence would exclude the addition of positively charged residues which normally complement the negatively charged Orai1 C-terminal helices, and we showed that the 2β insertion results in a decreased α-helicity. Thus, the 2β variation likely precludes Orai1 channel activation due to disruption of interactions with the Orai1 C-terminal cytosolic helices.

How does STIM2β inhibit SOCE? Given our data showing that the 2β insert does not prevent dimerization of OASF, heterodimerization of STIM2β with STIM2 and/or STIM1 is likely key for mediating an inhibitory effect. In the dimeric structure of CC1-CC2, both CC2 helices are required to form the pocket which binds two Orai1 C-terminal helices (Figure 8A,B). Hence, structural changes or unfolding induced by the 2β insert in one CC2 helix would collapse both Orai1 C-terminal helix binding sites if the perturbations extend in both directions along the helical axis. Therefore, we speculate that heterodimerization would attenuate the activation of Orai1 channels via a collapse of the Orai1 C-terminal helix binding pocket composed of both CC2 helices. In a passively mediated mechanism, inhibition could occur via sequestration of STIM1 and STIM2 molecules after heterodimerization; in an actively mediated mechanism, inhibition could occur via altered binding architecture with the Orai1 C-terminal domains due to a structurally distorted CC2-composed binding pocket.

It is important to note that, based on our work, the maximal Ca^2+^-binding induced conformational change in OASF is more marked in proteins containing the 2β insert. Thus, 2β appears to promote a conformation within OASF associated with Ca^2+^-dependent inactivation of Orai1 channels. Given that the ID is downstream of CC3, it is possible that 2β and Ca^2+^ binding to ID affect CC3 homomerization previously found to be vital in the activation of Orai1 channels [33,34]. Indeed, the Ca^2+^ binding-induced increases in aggregation observed here for all OASF proteins is consistent with a conformational change affecting the homomerizing CC3. Our observation that 2β causes a shift in the SEC-MALS elution profile (i.e., to earlier elution times) of STIM1-OASF, such that STIM1-2β-OASF matches STIM2-2β-OASF without any change in the dimer molecular weight, is similar to the effect of the L251S mutation which causes a conformational extension of STIM1-OASF in promoting Orai1 activation [27]. This conformational extension may more readily allow for Ca^2+^ interactions with the ID.

In conclusion, our study demonstrates that the 2β insert inhibits STIM activity by incorporation into the OASF structural context, independent of the STIM2-specific sequence of amino acids composing OASF. Given that STIM2 is a slower activator of SOCE and has a weaker efficacy of Orai1 activation, our work demonstrates that 2β does not preclude SOCE activation by further depotentiating weak STIM2-mediated Orai1 activation, but rather, 2β independently perturbs the ability of OASF to induce SOCE.

## 4. Methods

### 4.1. STIM OASF Expression and Purification

The human STIM2-OASF region (i.e., residues 238–495; NCBI Accession: NP_065911.3) was cloned into a pET-28a expression vector, which was previously used successfully for the expression of human STIM1-OASF [27,48,50]. The pET-28a vectors encoding STIM1-2β-OASF and STIM2-2β-OASF were generated via PCR-mediated site-directed mutagenesis to introduce the VAASYLIQ insert immediately following G379 and E383 codons in pET-28a-STIM1-OASF and -STIM2-OASF, respectively.

All STIM OASF proteins were expressed in BL21 (DE3) *E. coli* cells essentially as described previously for STIM1-OASF [27,48,50]. Briefly, the 6× His-tagged-OASF proteins were purified under denaturing conditions using guanidine-HCl. HisPur nickel-nitrilotriacetic acid resin (Thermo Scientific, Waltham, MA, USA) was used to selectively pull out the 6× His-tagged-OASF proteins from the crude cell lysates. The 6× His-tagged-OASF proteins were renatured at 4 °C using a buffer composed of 20 mM TRIS, 300 mM NaCl, and 1 mM DTT (pH 8.8). Subsequently, the 6× His-tags were cleaved using bovine thrombin (~1 U·mg^−1^ of protein; EMD Millipore, Burlington, MA, USA). The final purification step by SEC was performed with a Superdex 200 26/60 PG HiLoad (GE Healthcare, Chicago, IL, USA) column and a running buffer composed of 20 mM TRIS, 300 mM NaCl, 1 mM DTT, pH 8.0. STIM1-OASF, STIM1-2β-OASF, STIM2-OASF and STIM2-2β-OASF protein concentrations were estimated using ε_280nm_ extinction coefficients of 0.9478, 0.9629, 0.9948 and 1.0088 mg·mL^−1^·cm^−1^, respectively. Unless otherwise stated, the experimental buffer used in all experiments was identical to the running buffer.

### 4.2. Far UV CD Spectroscopy

Far-UV CD spectra were acquired as an average of 3 accumulations between 240 to 200 nm in 1 nm increments using the 0.1 cm pathlength quartz cuvette, 8 s averaging time at each wavelength, 1 nm bandwidth. Data were acquired at 0.2 mg·mL^−1^ protein at 20 °C using a Jasco J-815 CD spectrometer equipped with a Jasco PTC-423S temperature controller (Jasco Inc., Easton, MD, USA).

Thermal melts were acquired by monitoring the change in CD signal at 222 nm as a function of temperature also using a 0.1 cm pathlength quartz cuvette, an averaging time of 8 s at each temperature, 1 nm bandwidth and a 1 °C min^−1^ scan rate. The apparent midpoint of temperature denaturation (T_m_) was taken as the temperature showing a 50% relative change in ellipticity at 222 nm.

### 4.3. ANS Fluorescence Spectroscopy

Extrinsic ANS fluorescence was monitored using a Cary Eclipse spectrofluorimeter (Varian Inc., Palo Alto, CA, USA). Protein samples were prepared at a final concentration of 0.14 mg·mL^−1^ supplemented with 0.5 mM EDTA and 50 µM ANS (Sigma, St. Louis, MO, USA). ANS fluorescence emission spectra were acquired at 35 °C from 400 to 600 nm using an excitation wavelength of 372 nm. Control spectra were acquired in the absence of protein using identical settings and buffer conditions.

### 4.4. SEC-MALS

OASF proteins at 1 mg mL^−1^ protein were injected into a Superdex 200 Increase 10/300 GL gel filtration column (GE Healthcare) using the ÄKTA Pure system (GE Healthcare) housed at 10 °C. The column was connected in-line to a Dawn HELEOS II MALS detector equipped with a 662 nm laser source and Optilab T-rEX differential refractometer equipped with 658 nm LED source (Wyatt Technology, Santa Barbara, CA, USA). Protein concentrations through the eluted peaks were determined using a refractive index increment of dn·dc^−1^ = 0.185 mL·g^−1^. Molecular weights were calculated by Zimm plot analysis using the ASTRA software (v6.1.5.22; Wyatt Technology).

### 4.5. DLS

DLS experiments were performed on a DynaPro Nanostar (Wyatt Technology) equipped with a 658 nm laser. Protein samples were prepared by centrifuging 0.5 mg·mL^−1^ protein solutions at 12,000× *g* for 3 min to remove large particulates, before a 5 µL aliquot was loaded into a dust-free quartz MicroCuvette (JC501). All DLS acquisitions were performed at 37 °C, where the protein sample was allotted 5 min of equilibrium time prior to acquiring 10 consecutive readings averaged over 5 s each. Distributions of hydrodynamic radii were extracted from the autocorrelation function using the regularization algorithm built into the accompanying instrumental software (Dynamics v7; Wyatt Technology).

### 4.6. SAXS

Protein concentrations of 1.041, 0.855, 1.045 and 1.109 mg·mL^−1^ were used for STIM1-OASF, STIM1-2β-OASF, STIM2-OASF and STIM2-2β-OASF, respectively. SAXS profiles for the protein and matching buffer controls were performed in a 1 mm quartz cuvette (50 µL) at 4 °C using a 3 h X-ray exposure time (180 × 1 min frame). An aligned SAXSpace instrument (Anton PaarGmbH, Graz, Austria) working in line collimation mode was used to carry out the assessment. The SAXSpace X-ray generator contained a long-fine focus glass sealed copper tube (40 kV/50 mA, wavelength = 0.1542 nm), and the detector used was a Mythen2 R 1K 1D detector (DECTRIS Ltd., Baden, Switzerland). Temperature was controlled using a Peltier-controlled TCStage150.

*R_g_* was calculated as √(−3 × slope) of the Guinier plots. D_max_ was determined from the pair distance-distribution functions [P(*r*)], initially estimated using DATGNOM [55], and then manually adjusted until a gradual decay to zero was observed in the P(*r*) plots. The D_max_ was used in ab initio shape reconstruction using DAMMIF [43]. Thirty DAMMIF ab initio structures were determined for each OASF protein. These thirty structures were clustered and averaged using DAMCLUST [56].

### 4.7. Fura-2 Fluorimetry

For functional assessment of SOCE, we used HEK293 cells stably expressing YFP-Orai1. These cells were lifted off 10 cm culture dishes by gentle pipetting and transferred into a 15 mL conical tube. The resultant cell suspension was supplemented with 2 µM Fura-2-AM (Alfa Aesar, Haverhill, MA, USA) and incubated in the dark at 37 °C for 45 min. Subsequently, the cells were pelleted by centrifugation and washed with 15 mL of HEPES buffered saline solution (HBSS; 140 mM NaCl, 4.7 mM KCl, 1.13 mM MgCl, 10 mM glucose, and 10 mM HEPES, pH 7.4). Finally, the cells (~5 × 10^6^) were resuspended in 1.2 mL of the HBSS buffer and transferred into a quartz cuvette.

Fura-2 fluorescence was assessed after the addition of 0.5 mM ethylene glycol tetraacetic acid (EGTA) and allowing the cuvette containing the cell suspension to equilibrate for 3 min at 22.5 °C. Fluorescence (F) using alternating excitation wavelengths = 340 and 380 nm and a constant emission wavelength = 510 nm was monitored for 1000 s total using a Cary Eclipse spectrofluorimeter (Varian Inc., Palo Alto, CA, USA). At 100 s, 2 µM TG was added to the cell suspension. At 600 s, 2.5 mM CaCl_2_ (i.e., 2.0 mM net CaCl_2_) was added to the cell suspension. The data were plotted as a normalized F/F_0_ ratio, where F_0_ was taken as the average of the first 100 data points.

## Figures and Tables

**Table 1:** Summary of STIM OASF SEC-MALS data.

Protein	Ca^2+ (a)^	Elution Volume (mL) ^(b)^	Molecular Weight (kDa) ^(c)^	Stoichiometric Ratio ^(d)^
STIM1-OASF	−	13.59 ± 0.14	67.6 ± 1.4	2.18
STIM1-2β-OASF	−	12.69 ± 0.05	64.9 ± 1.7	2.02
STIM2-OASF	−	12.69 ± 0.02	61.4 ± 1.3	1.98
STIM2-2β-OASF	−	12.85 ± 0.02	60.8 ± 0.2	1.96
STIM1-OASF	+	13.55 ± 0.01	62.3 ± 3.7	2.00
STIM1-2β-OASF	+	13.03 ± 0.30	63.0 ± 1.3	1.96
STIM2-OASF	+	12.76 ± 0.08	61.8 ± 2.1	1.99
STIM2-2β-OASF	+	12.87 ± 0.20	61.3 ± 1.6	1.97

^(a)^: SEC-MALS conducted in the absence (−) and presence (+) of 25 mM CaCl_2_. ^(b)^: Elution volumes on the Superdex 200 Increase 10/300 GL (GE Healthcare). ^(c)^: MALS molecular weights averaged under the major portion of the elution peak. ^(d)^: Stoichiometric ratios taken as the MALS molecular weight/theoretical monomer masses.

## Supplementary Materials

Supplementary materials can be found at http://www.mdpi.com/1422-0067/19/11/3316/s1.
