# Supplementary material for: The 2β Splice Variation Alters the Structure and Function of the Stromal Interaction Molecule Coiled-Coil Domains
Source: Int J Mol Sci. 2018 Oct 25;19(11):3316. doi: 10.3390/ijms19113316 (PMC6274866; doi:10.3390/ijms19113316)
Supplement: Supplementary file 1 [file ijms-19-03316-s001.zip › ijms-375938 Supplementary Materials for proof.docx]

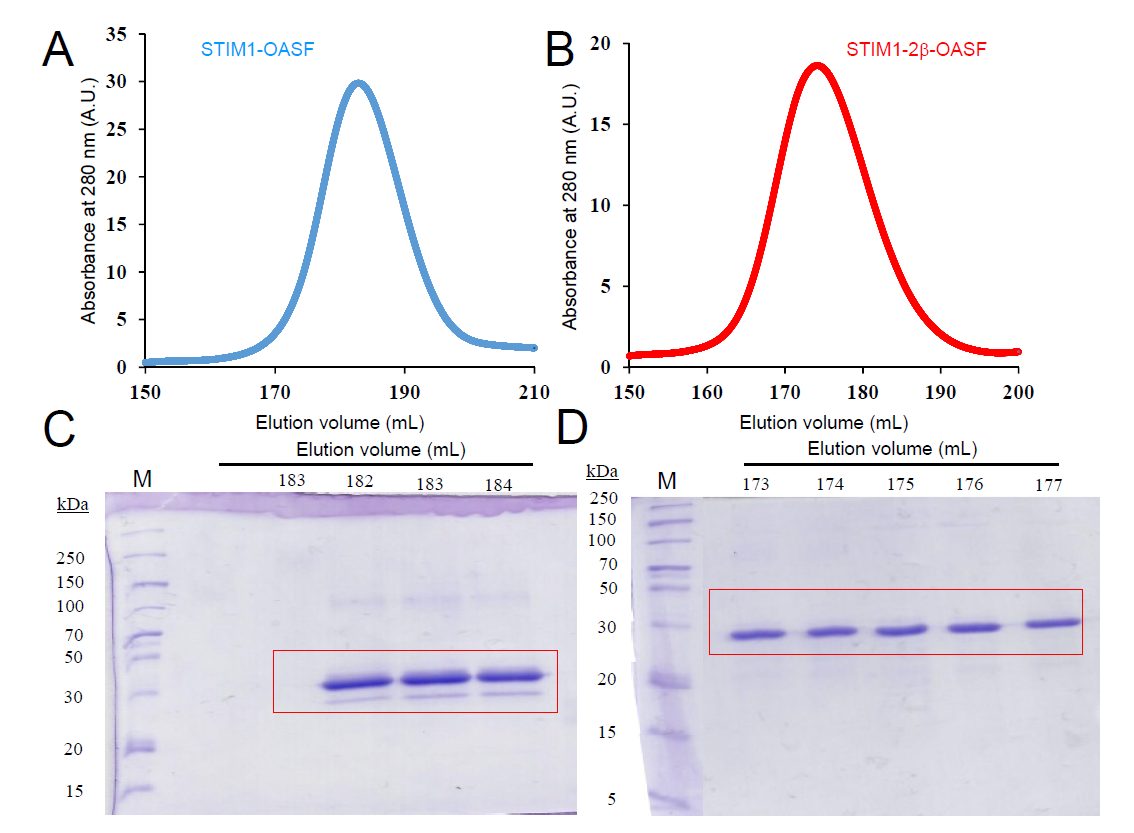


**Figure S1.** Expression and purification of STIM1 OASF proteins. SEC elution profile of STIM1-OASF (**A**) and STIM1-2β-OASF (**B**). Proteins were purified using a Superdex 200 26/60 column (GE Healthcare). Coomassie blue R-250 staining of STIM1-OASF (theoretical monomer weight of 31 kDa) (**C**) and STIM1-2β-OASF (theoretical monomer weight of 32 kDa) (**D**) separated on a 15% (w/v) SDS-PAGE gel. The red boxes highlight the purified OASF proteins.


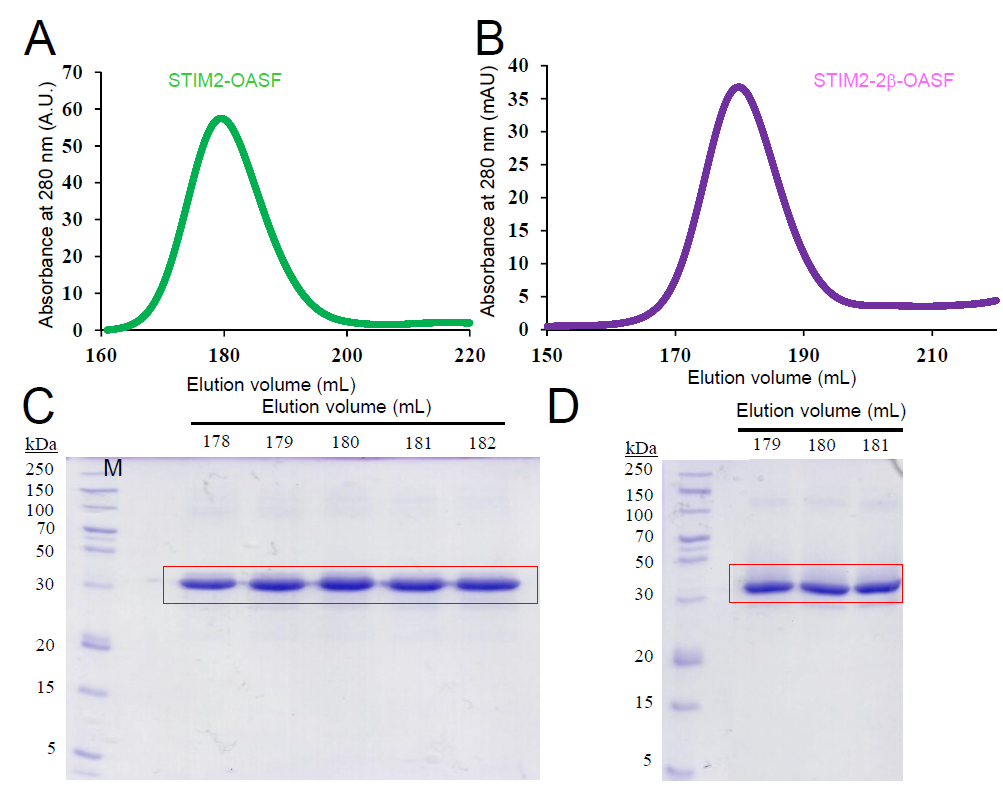


**Figure S2.** Expression and purification of STIM2 OASF proteins. SEC elution profile of STIM2-OASF (**A**) and STIM2-2β-OASF (**B**). Proteins were purified using a Superdex 200 26/60 column (GE Healthcare). Coomassie blue R-250 staining of STIM2-OASF (theoretical monomer weight of 31 kDa) (**C**) and STIM2β-OASF (theoretical monomer weight of 32 kDa) (**D**) separated on a 15% (w/v) SDS-PAGE gel. The red boxes highlight the purified OASF proteins.


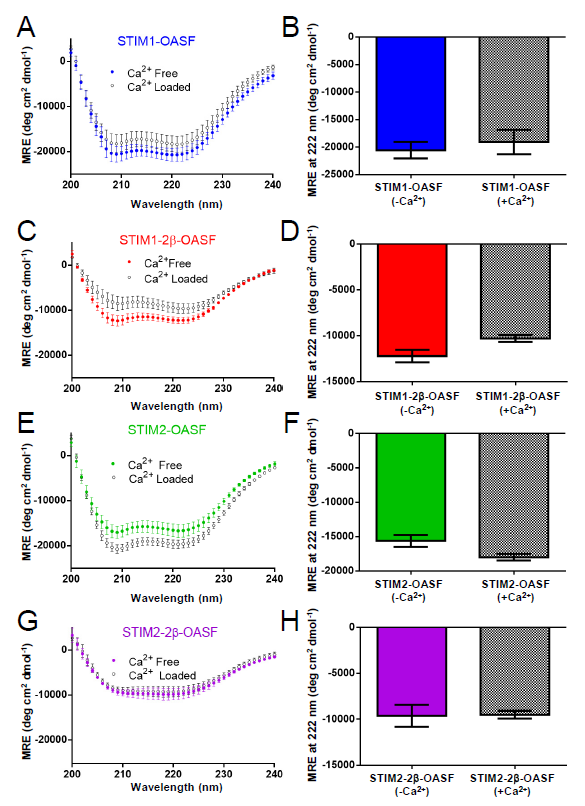


**Figure S3.** Ca^2+^ sensitivity of STIM OASF secondary structure. Far-UV CD spectra of STIM1-OASF (**A**), STIM1-2β-OASF (**C**), STIM2-OASF (**E**) and STIM2-2β-OASF (**G**) in the absence (coloured symbols) and presence of 25 mM CaCl_2_ (open circles). Comparison of STIM1-OASF (**B**), STIM1-2β-OASF (**D**), STIM2-OASF (**F**) and STIM2-2β-OASF (**H**) MRE at 222 nm. Spectra were acquired using 0.2 mg mL^−1^ protein at 20 °C. Data are means ± SEM of *n* = 3 separate protein purifications.


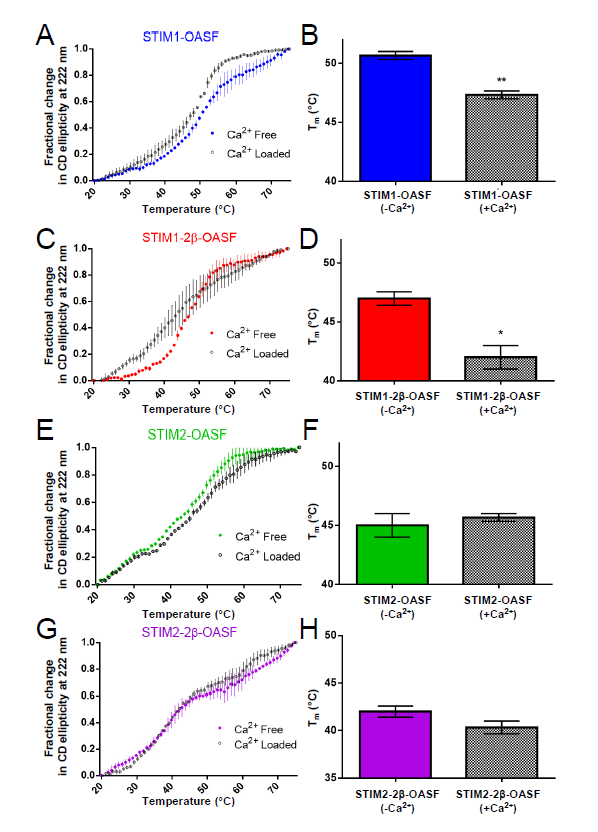


**Figure S4.** Ca^2+^ sensitivity of STIM OASF thermal stability. Thermal unfolding profiles of STIM1-OASF (**A**), STIM1-2β-OASF (**C**), STIM2-OASF (**E**) and STIM2-2β-OASF (**G**) in the absence (coloured symbols) and presence of 25 mM CaCl_2_ (open circles). Comparison of STIM1-OASF (**B**), STIM1-2β-OASF (**D**), STIM2-OASF (**F**) and STIM2-2β-OASF (**H**) T_m_ values extracted from the thermal melt acquired in the absence (coloured circles) and presence of 25 mM CaCl_2_ (open circles). Thermal melts were acquired using 0.2 mg mL^−1^ protein. Data are means ± SEM of *n* = 3 separate protein purifications. In *B* and *D*, **p* < 0.05 and ***p* < 0.01 using Student’s *t*-test.


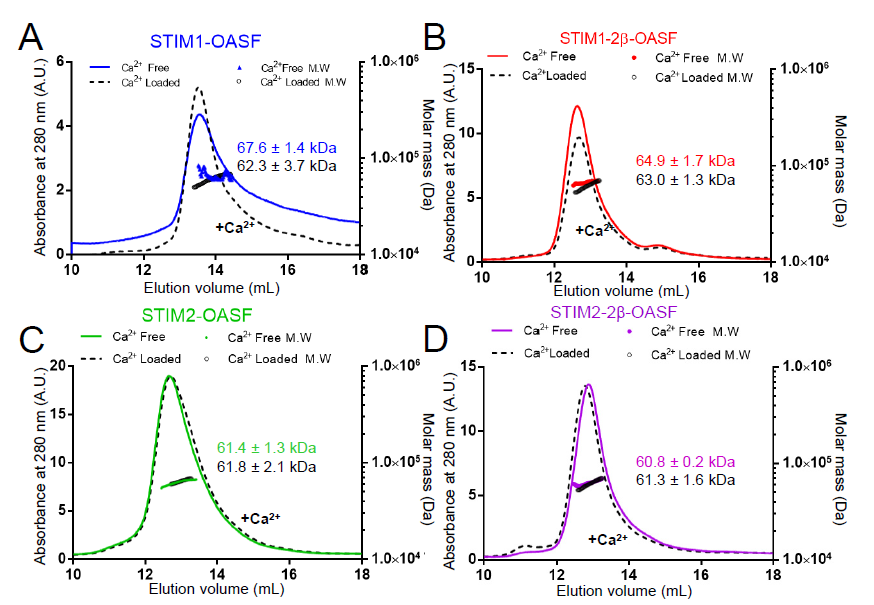


**Figure S5.** Ca^2+^ sensitivity of STIM OASF quaternary structure. SEC-MALS elution profiles and molecular weight determinations for STIM1-OASF (**A**), STIM1-2β-OASF (**B**), STIM2-OASF (**C**), and STIM2-2β-OASF (**D**). The elution profiles acquired in the presence of 25 mM CaCl_2_ are shown as dashed black lines in each panel. The MALS-determined molecular weights are shown under the Ca^2+^-free peaks (coloured circles) and Ca^2+^-loaded peaks (closed black circles). SEC-MALS was performed with 100 µL injections of ~1 mg mL^−1^ protein using a Superdex 200 Increase 10/300 GL column housed at 10 °C.


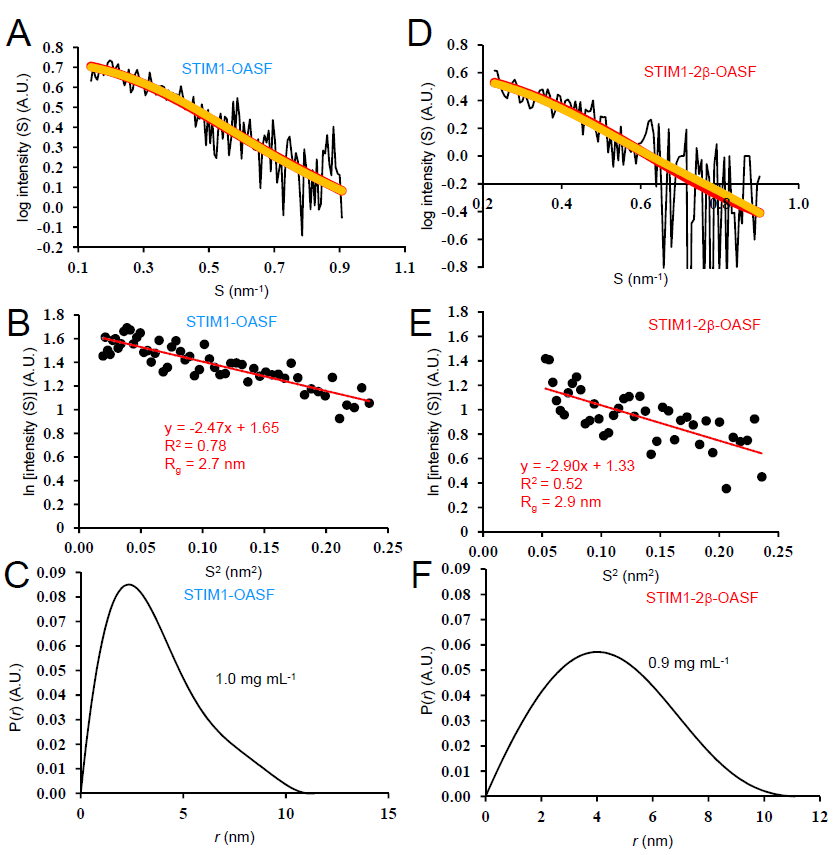


**Figure S6.** Conformational analysis of STIM1 OASF proteins. (**A**) SAXS scattering profile of STIM1-OASF. (**B**) Guinier plot for R_g_ determination of STIM1-OASF. (**C**) Porod distance distribution functions of for D_max_ determination of STIM1-OASF. (**D**) SAXS scattering profile of STIM1-2β-OASF. (**E**) Guinier plot for R_g_ determination of STIM1-2β-OASF. (**F**) Porod distance distribution functions for D_max_ determination of STIM1-2β-OASF. In *A* and *D*, the scattering profiles reconstructed from the Porod distribution plots are shown in red while the profiles reconstructed from the DAMMIF models (see Figure 6) are shown in yellow. Data were acquired at 4 °C.


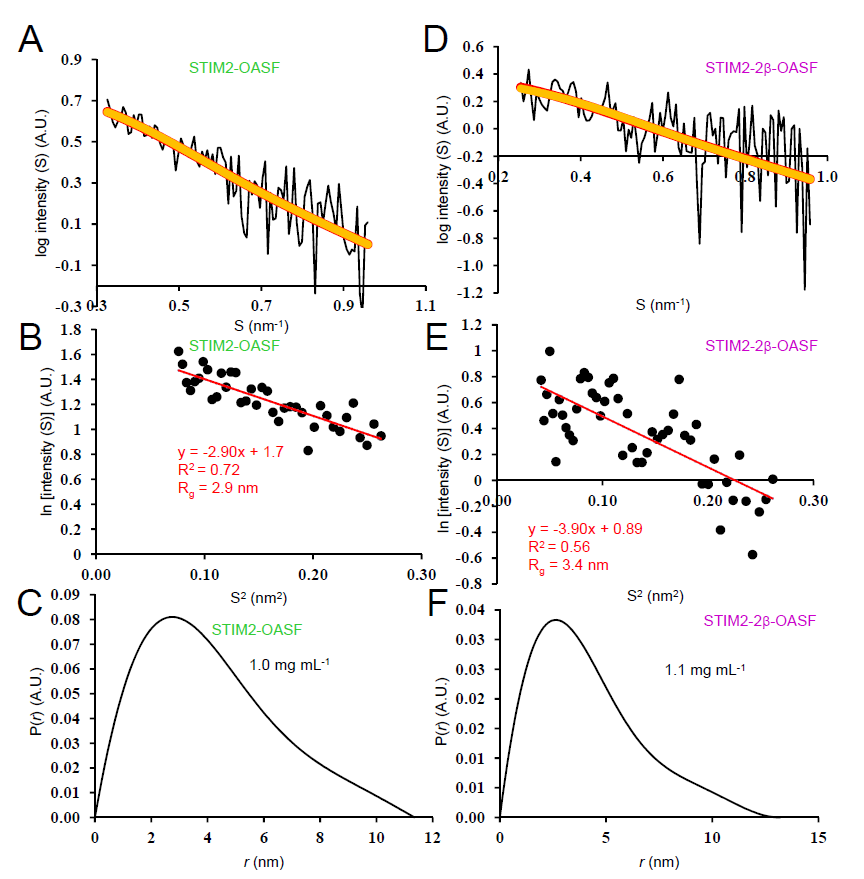


**Figure S7.** Conformational analysis of STIM2 OASF proteins. (**A**) SAXS scattering profile of STIM2-OASF. (**B**) Guinier plot for R_g_ determination of STIM2-OASF. (**C**) Porod distance distribution functions for D_max_ determination of STIM2-OASF. (**D**) SAXS scattering profile of STIM2-2β-OASF. (**E**) Guinier plot for R_g_ determination of STIM2-2β-OASF. (**F**) Porod distance distribution functions for D_max_ determination of STIM2-2β-OASF. In *A* and *D*, the scattering profiles reconstructed from the Porod distribution plots are shown in red while the profiles reconstructed from the DAMMIF models (see Figure 6) are shown in yellow. Data were acquired at 4 °C.
